# Cell Cycle Regulation in the Plant Response to Stress

**DOI:** 10.3389/fpls.2019.01765

**Published:** 2020-01-30

**Authors:** Feifei Qi, Fuxin Zhang

**Affiliations:** Shandong Provincial Key Laboratory of Animal Resistance Biology, Institute of Biomedical Sciences, College of Life Sciences, Shandong Normal University, Jinan, China

**Keywords:** stress response, cell cycle, cyclin, cyclin-dependent kinase, immunity

## Abstract

As sessile organisms, plants face a variety of environmental challenges. Their reproduction and survival depend on their ability to adapt to these stressors, which include water, heat stress, high salinity, and pathogen infection. Failure to adapt to these stressors results in programmed cell death and decreased viability, as well as reduced productivity in the case of crop plants. The growth and development of plants are maintained by meiosis and mitosis as well as endoreduplication, during which DNA replicates without cytokinesis, leading to polyploidy. As in other eukaryotes, the cell cycle in plants consists of four stages (G1, S, G2, and M) with two major check points, namely, the G1/S check point and G2/M check point, that ensure normal cell division. Progression through these checkpoints involves the activity of cyclin-dependent kinases and their regulatory subunits known as cyclins. In order for plants to survive, cell cycle control must be balanced with adaption to dynamic environmental conditions. In this review, we summarize recent advances in our understanding of cell cycle regulation in plants, with a focus on the molecular interactions of cell cycle machinery in the context of stress tolerance.

## Introduction

All organisms have evolved mechanisms to cope with a broad range of biotic and abiotic stresses (Hou et al., 2012; Liu et al., 2012; Tian et al., 2017; Chang et al., 2018). The former is caused by external organisms such as bacteria, virus, fungi, and animals (Liang et al., 2010; Huang et al., 2013; Zhang et al., 2014; Zhang F. et al., 2015; Xing et al., 2017), whereas the latter is attributable to environmental conditions, such as drought (Zhao S. et al., 2016; Tang et al., 2017), cold temperature (Sui, 2015), changes in salinity (Cui et al., 2018), and so on. Unlike animals, plants are sessile and have therefore developed unique stress responses involving many types of sensor that ensure their survival.

In nature, plants are vulnerable to pathogens and predators. The immune system in animals has innate and adaptive components; immune signaling cascades have been widely studied in fish (Liu et al., 2011; Sun et al., 2012; Li et al., 2013; Liu T. et al., 2017), cow (He C. et al., 2016; Hou et al., 2017; Hou et al., 2018), pig (Zheng S. et al., 2017; Zheng et al., 2018), and mouse (Cui et al., 2011; Yoshida et al., 2019). In contrast, plants lack the adaptive component and rely primarily on two layers of innate immunity in their response to pathogen infection. Plants sense pathogens through recognition of conserved microbe-associated molecular patterns and host-derived damage-associated molecular patterns (Boller and Felix, 2009). Additionally, disease resistance proteins recognize effector molecules secreted by pathogens and induce a more robust and rapid immune response that usually leads to programmed cell death (Zhang et al., 2018). However, immune activation perturbs the cell cycle, which controls plant growth; recent evidence suggests a close connection between cell cycle kinetics and immunity in plants (Bao and Hua, 2015).

Plants can detect various environmental signals such as high salt concentration (Shen et al., 2014; Wang S. et al., 2014; Liu S. et al., 2017; Sui et al., 2017), cold temperature (Tang et al., 2012; Liu et al., 2013; Zhao X. et al., 2014; He Y. et al., 2016), reactive oxygen species levels (Li K. et al., 2012), waterlogging (Song et al., 2011; Chen et al., 2016; Zhang Y. et al., 2017), phosphate scarcity (Sui et al., 2017), and ultraviolet (UV) radiation (Chen et al., 2013; Deng et al., 2015). Failure to appropriately respond to these stressors decreases plant productivity and affects human food and animal feed supplies. As such, considerable effort has been invested in developing methods to improve stress resistance in crop plants such as wheat (Gong et al., 2017; Kong et al., 2017), peanut (Hou et al., 2014; An et al., 2015), rice (Yang et al., 2013; Bai et al., 2016), tobacco (Guo et al., 2013; Cao et al., 2017), tomato (Sun et al., 2010; Meng et al., 2015), maize (Zhao K. et al., 2010; Wang S. et al., 2014), cotton (Zhao et al., 2012; Wang et al., 2016), and bean (Weng et al., 2011; Zhao B. et al., 2016).

**Figure 1:**
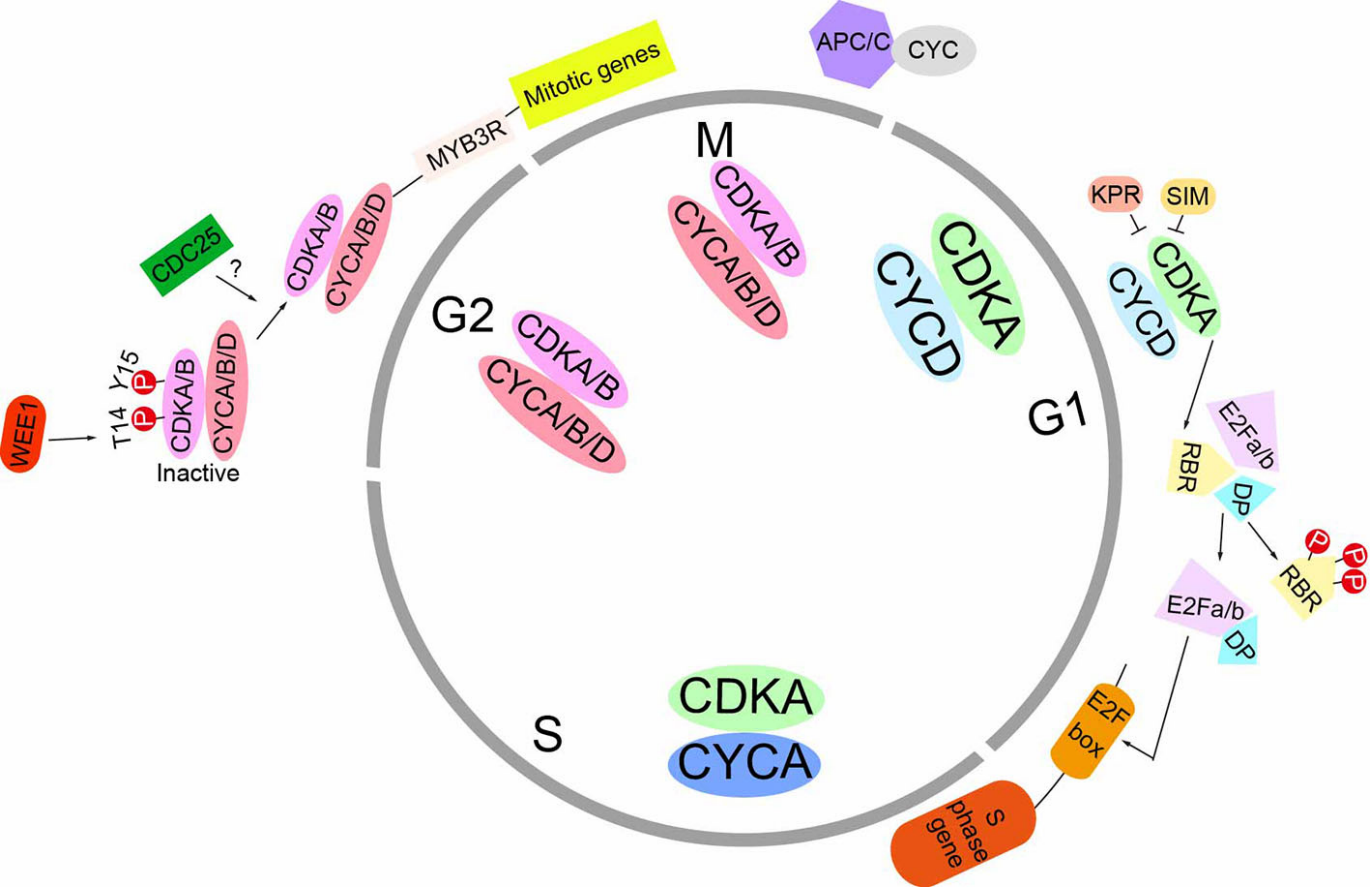
Schematic representation of the mitotic cell cycle in plants. At the G1 phase, D-type cyclins (CYCD) interact with the A-type CDK (CDKA), forming the CDKA/CYCD complex. The activity of CDKA/CYCD complex can be negatively regulated by KPR and SIM proteins. Once activation, this complex phosphorylates RBR to release the transcript factor E2Fa/b-DP. This E2Fa/b-DP complex binds to the E2F box and activate the transcription of S phase genes. At the G2 and M phase S, *CYCA* and *CYCB* are strongly expressed and their gene products assemble with CDKA and CDKB. The CYCD can also associate with CDKs. At the beginning of G2 phase, CDK activity are inhibited because of the phosphorylation of Y14 and T15 site by WEEI kinase. The CDC25-related kinase, which removes the inhibitory phosphate groups, still needs to be identified. Once the CDK/CYC complex are active, they phosphorylate MYB3R transcription factors and activate mitotic genes’ transcription. Mitotic exit requires anaphase-promoting complex (APC), which degrades cyclins through ubiquitin-proteasome pathway.

Plants growth results from the coordinated interaction of mitotic cell cycle and cell expansion. As in animals, the plant cell cycle consists of four distinct phases: G1 (postmitotic interphase), S (DNA synthesis phase), G2 (premitotic interphase), and M (mitosis/cytokinesis). Cell cycle progression is driven by cyclin-dependent kinases (CDKs) which cooperated with cyclins. There are seven classes of cyclins in plants which consists of approximately 60 cyclin genes. Among them, we know more about the A, B, and D classes. In general, D-type cyclins are considered as the regulators of G1-to-S transition, A-type cyclins are thought to control S-to-M phase, and B-type cyclins control G2-to-M transition. At G1-to-S transition, CDKA/CYCD complex phosphorylates the retinoblastoma-related (RBR) protein which activates the S-phase transcription factor, “E2F”. E2Fs promotes the G1–S transition by modulating the expression of genes involved in DNA replication, cell-cycle progression and chromatin dynamics (del Pozo et al., 2006). The negative regulation of G1/S transition is the KRPs (Kip-related proteins) and SIM (SIAMESE) which play roles as the inhibitors of CDKA/CYCD complex. At G2/M transition, CDKA and CDKB bind to CYCA, CYCB, or CYCD and drive cells into division. Meanwhile, the activities of CDKA and CDKB are negatively regulated by the WEE1 kinase. The CDC25 homologous kinase, which dephosphorylates the inhibitory phosphorylated site in CDK, still needs to be identified. Once the CDK/CYC complexes are activated, they trigger the G2-to-M transition through phosphorylating numerous of different substrates. Mitotic exit requires the proteolytic degradation of the cyclin subunits which mediated by the anaphase-promoting complex (APC) (Capron et al., 2003) (**Figure 1**). Although the basic mechanisms which control mitotic cell cycle progression are conserved from plants to vertebrate, plants have unique molecules orchestrating the cell cycle. Moreover, plants have usually a variety of cyclins even though the function of many of which is unclear. At the same time, plants don’t have Cyclin E, which is a major target for organ regulators in animals (Halder and Johnson, 2011).

In addition to mitotic cell cycle, the endocycle plays pivotal role in plant growth. Endocycle means that DNA replication in the absence of cytokinesis and leads to polyploid. The switch from the mitotic cell cycle to the endocycle involves changes in the regulation and abundance of a wide broad of cyclin-dependent kinases (CDKs), cyclins (CYC), and regulatory proteins/transcription factors. Endopolyploidy is common in reproductive tissues of plants, for example the nutritive tissue of the endosperm in seeds. The onset of endopolyploidy is induced in some cell tissues in response to stress. In this review, we summarize the molecular link between environmental signals and cell cycle progression and the stress response in plants.

### Cell Cycle Regulation and Immunity

All animals and plants are vulnerable to infection with viruses and bacteria. For example, cattle and pigs are easily infected with bovine ephemeral fever virus and porcine circovirus, respectively (Yu et al., 2012; Wang X. et al., 2014), and rely on innate and adaptive immunity for protection. The bacterial strain BGI-1, isolated from the gut of the German cockroach (*Blattella germanica*), contributes to anti-entomopathogenic fungal infection (Huang et al., 2013). Additionally, pathogenic microorganism invasion induces the innate immune responses in zebrafish (Yang H. et al., 2017). Toll-like receptor (TLR)22 (Li H. et al., 2017), TLR18 (Shan et al., 2018), X box-binding protein 1 (Li T. et al., 2017), and interferon regulatory factor 1 (Shan et al., 2016; Zhu et al., 2016a; Zhu et al., 2016b) have been identified as critical molecules involved in immunity in the common carp. However, unlike animals, plants lack a somatic adaptive immune system and instead rely on the innate immunity of each cell to detect signals originating from sites of infection (Ausubel, 2005; Chisholm et al., 2006). Plants detect the extracellular pathogens through plasma membrane-localized pattern recognition receptors (PRRs) that recognize conserved microbe-associated molecular patterns (MAMPs), such as bacterial flg22 and elf18 (active epitopes of bacterial flagellin and elongation factor-Tu, respectively), and then drive patterns-triggered immunity (PTI) (Petutschnig et al., 2010). In addition, plants possess a variety of resistance (R) genes which encode proteins containing nucleotide binding (NB) and leucine rich repeat (LRR) domains. NB-LRR proteins induce effector-triggered immunity (ETI) after specific perception of pathogenic T3SEs.

Interaction with pathogens influences cell cycle progression in plants. In *Arabidopsis thaliana*, Cabbage leaf curl virus infection alters the expression of cell cycle-related factors; overexpression of CYCD3;1 or E2FB, both of which promote mitosis, increases polyploidy and strongly inhibits Cabbage leaf curl virus infection (Ascencio-Ibanez et al., 2008). Powdery mildew (PM), which is the main pathogen of cereal crops, stimulates the endocycle and increases calcium signaling at the infection site in *Arabidopsis*. The loss-of-function mutant of MYB3R4, a transcription factor required for endocycle initiation, suppresses PM growth (Chandran et al., 2009). However, it’s currently unclear how cell immunity response is communicated to cell cycle progression.

In a broad sense, PTI functions through several pathways such as the hormone pathways or the phosphorylation of mitogen-activated protein kinases (MAPKs) or the rapid production of reactive oxygen species (ROS) and Ca^2+^-mediated activation of Ca^2+^-dependent kinases (CDPKs) that activate transcription factors to induce the expression of down-stream genes. Immunity-triggered growth inhibition might be partly related to jasmonates (JA)-mediated inhibition of the cell cycle. Increased JA level is a positive defense response to pathogen infection. Chen et al. (2011) indicated that JA suppressed *CDKA;1* and *CYCB1;1*. Recent studies have further revealed that JA signaling stabilizes DELLA proteins which induce the CDKA and CDKB inhibitors KRP2, SIM, and SMR (Achard et al., 2009). Therefore, inhibited cell cycle progression may be a consequence of hormone. Harnessing the toxic properties of reactive oxygen species (ROS) to fight against invading pathogens might be another reason for prolonged cell cycle. ROS in adequate concentration directs the invaded plant cells towards apoptosis so as to restrict the fungal infection spread (Bastas, 2014). In mammalian cells, ROS influence cell cycle progression *via* phosphorylation and ubiquitination of CDKs and cell cycle regulatory molecules, such as CKI and Cdc25. ROS exert their effect on Cdc25 activity *via* enhancing phosphorylation of Cdc25 or alternatively inactivation of Cdc25 by sulfonation of cysteine in the active site (Verbon et al., 2012). Therefore, it’s reasonable to speculate plant ROS play roles in the similar ways as in mammalian. Regardless of pathogen type, ETI is commonly accompanied by programmed cell death (PCD) at the infection site to stop pathogen spread (Zebell and Dong, 2015).

It’s of significant importance to investigate whether cell cycle regulatory genes promote immunity positively. The *Arabidopsis* genes *OMISSION OF THE SECOND DIVISION* (*OSD*)*1* and its homolog *UV-B-INSENSITIVE* (*UVI*)*4* negatively regulate the activities of anaphase-promoting complex (APC)/cyclosome. Overexpression of both *OSD1* and *UVI4,* or *APC6* deficiency, enhances immunity to pathogens by stimulating the expression of disease resistance genes, such as *SNC1*, which is associated with inhibition of the endocycle (Bao et al., 2013). However, the mechanisms by which *OSD1* and *UVI4* enhance immunity still need to be explored. In fact, recent studies indicated a direct link of cell cycle regulators in immunity, for example, *TEOSINTE BRANCHED1, CYCLOIDEA, PCF1* (*TCP*)*15,* and *MODIFIER OF SNC1* (*MOS1*). As a transcription factor, TCP15 regulates the transcription of *CYCA2;3* (Li Z. et al., 2012) and *SUPPRESSOR OF rps4-RLD1*, which encodes a protein that negatively regulates plant immunity (Kim et al., 2014). In *Arabidopsis*, MOS1 directly interacts with TCP15, and affect the expression of *CYCD3;1* and the immune receptor gene *SUPRESSOR OF npr1-1*, *CONSTITUTIVE* (*SNC*)*1*. Additionally, *CYCD3;1* overexpression enhances immune responses and SNC1 protein levels. Moreover, G1-S phase checkpoint proteins Rb and E2F engaged in immune-related PCD (Chandran et al., 2014).

Taken together, these findings indicate that plants balance cell cycle regulation and immune response through manipulating the level of cyclins or the activation of CDKs. Plants respond to pathogen attack *via* arrested cell cycle progression depend on the state at which plants are attacked. At mitosis, inhibition of APC/C activation will contribute to immunity. At G1, S, or G2 phase, operating the expression of cyclins or check point proteins, such as E2Fs and MYB3R, are of good benefit to immunity. It’s reasonable to speculate that the biological significance of using cell cycle components as regulators of immunity is to turn cells from endoreduplication, which favors pathogen infection, to cell cycle arrest or cell death, and therefore prevent pathogen infection spread. Though recent studies have broadened our knowledge of immunity-cell cycle trade-off, future efforts are needed to identify the immunity-growth pathway and the detailed molecular mechanisms. Single-cell RNA sequence technology, RNA-Seq approach and proteomic analysis will help to underscore molecular connections that ensure antagonistic regulation of growth and immunity in plants under natural or abiotic stress conditions.

### Cell Cycle Regulation and Abiotic Stress Adaptation

Salinity, temperature, moisture, and light are abiotic factors that influence plant growth and crop yield, which is shown to be a consequence of cell proliferation and the cell expansion. A rapid response to changing conditions is critical for successful adaptation. Plants respond to different stresses through a variety of mechanisms. In the following sections, we highlight the role of protein kinases that regulate cell cycle progression in the response to abiotic stressors.

#### Salt Stress

In general, high salinity leads to ion toxicity, water deficit, and oxidative stress. Osmotic stress also reduces cell division rates and cell numbers in leaves, roots, or the shoot meristem. Calcium ion (Ca^2+^) responses, reactive oxygen species (ROS) burst and abscisic acid (ABA)-dependent protein kinases are involved in response to high salinity. However, recent evidence has begun to underline the cell cycle regulator’s importance in stress response, especially cyclins and CDKs. *A. thaliana*, a non–salt-tolerant plant, is widely used as a model to investigate the molecular mechanisms of salt tolerance (Qi et al., 2010; Sun et al., 2013; Zhang L. et al., 2017; Zheng Y. et al., 2017). Two CDKs have been identified in *A. thaliana*: *AtCDC2a* regulates the G1/S and G2/M transitions, whereas *AtCDC2b* is mainly expressed in S and G2 phases in meristem tissues. Upon NaCl treatment, the transcription of *AtCDC2a* and *CYCA2;1* was decreased in the vascular cylinder of the root, which correlated with reduced lateral root formation. In root tips, *AtCDC2a*, *CYCA2;1*, and *CYCB1;1* were downregulated along with reduced root growth (S et al., 2004; West et al., 2004). West et al. (2004) demonstrated that, in *Arabidopsis thaliana* roots, mild salt stress leads to loss of CDK activity and reduced promoter activity of *CYCB1;2*. Severe salt stress transiently decreases the expression levels of the cyclins *CYCA2;1* and *CYCB;1*. In addition, Zhao L. et al. (2014) revealed that heat and cold treatments both induced a pronounced cell accumulation in G2/M transition and NaCl treatment results in an extensive inhibition in both S and G2/M phase by analyzing DNA content in maize (*Zea MAYS l*). Furthermore, the perturbed cell cycle progression is attributed to the dynamic histone acetylation change which participated in the control of CDKs and cyclins transcription. Thus, high salinity affects cell cycle regulation *via* control the cell cycle regulators. However, molecular players which perceive Na^+^ ions and confer salt stress to regulated cyclins and CDKs are still need to be unraveled.

Interestingly, there exists a group of salt-tolerant plants which grow in highly saline soil or water called halophyte (Zhao S. et al., 2010; Zhao et al., 2011; Zhang T. et al., 2015). An example of a halophyte is *Suaeda salsa* (Chen et al., 2010; Cheng et al., 2014; Guo et al., 2015; Guo et al., 2018), which produces dimorphic seeds on the same plant, brown seeds but not black seeds are able to germinate under conditions of high salinity owing to the presence of a seed coat (Wang et al., 2015; Song et al., 2016; Xu et al., 2016; Song et al., 2017). In this species, ion transport signaling contributes to salt tolerance through the action of various genes including *HIGH-AFFINITY POTASSIUM TRANSPORTER* (*SsHKT*)*1* (Shao et al., 2014), *SODIUM/HYDROGEN EXCHANGER* (*SsNHX*)*1* (Liu et al., 2018), and *ASCORBATE PEROXIDASE* (*SsAPX*) (Song and Wang, 2015). Besides, *S. salsa* treated with 200 mM NaCl generated healthier seeds with higher starch, soluble sugar, protein, and lipid contents than those treated with other concentrations of NaCl (Sui et al., 2010; Yang et al., 2010; Zhang et al., 2010; Li X. et al., 2012; Sun et al., 2015; Zhou et al., 2016). Another salt-tolerant plant is *Limonium bicolor*, a recretohalophyte with salt glands and a bladder that enable the secretion of excess salt into the environment (Yuan et al., 2013; Yuan et al., 2014; Yuan et al., 2015a; Yuan et al., 2015b; Yuan et al., 2016a; Yuan et al., 2016b). Recent studies show that the rate of secretion is determined by K^+^ and Ca^2+^ concentration (Ding et al., 2010; Feng et al., 2014; Feng et al., 2015; Leng et al., 2018). In *Thellungiella salsuginea*, microRNAs and unsaturated fatty acids enhance resistance to high salinity (Zhang et al., 2013; Sui and Han, 2014). It’s meaningful to detect whether the genes or microRNAs or unsaturated fatty acids mentioned above help to sustain the normal expression of cyclins and the activities of CDKs to some extent.

In conclusion, halophytes have evolved functional adaptations, ensuring their survival in saline environment. Analysis the difference between halophytes and glycophytes in transcriptomics and proteomics contributes to discover genes which is responsible for salt tolerance. Moreover, although most crop plants are glycophytes and are unable to tolerate high levels of salt stress, the abilities of glycophytes to withstand salt stress differ. This natural variation can be utilized to identify genetic components underlying salinity tolerance in glycophytes. Furthermore, screening proteins interacting with cyclins or CDKs under normal or stressed conditions are conductive to find molecules which confer salt stress signal to manipulated cell cycle regulators. Crops can be genetically engineered to resist high salinity through the expression of salt-responsive genes from halophytes; this has been demonstrated by the generation of salt-tolerant varieties of tomato, wheat, soybean, tobacco, peanut, cotton, and *A. thaliana* (Kong et al., 2011; Deng et al., 2016; Yang Z. et al., 2017).

#### Temperature Stress

Plants are continually exposed to changes in the ambient temperature. Both chilling and hot can negatively affect plant growth and development. Temperature stress triggers Ca^2+^ fluxes, kinase cascades, and accumulation of abscisic acid. These factors activate cell cycle checkpoints that delay entry into mitosis (Kadota et al., 2004; Kadota et al., 2005). In plants, more and more evidence has emerged to reveal the molecular mechanisms that regulate CDK functions in adapt to temperature stress. Under mild heat stress, proliferating cells are transiently arrested at G1/S or G2/M phase in BY2 cells, depending on the stage at which the stress was applied (Jang et al., 2005). The arrested cell cycle partially due to the reduced transcription of *CYCA* and *CYCB* and, as a consequence, decreased CDK activity. On the other hand, high temperatures induce a signaling cascade that leads to programmed cell death (Larkindale and Knight, 2002; Vacca et al., 2004).

Rymen et al. (2007) demonstrated that the increased cell cycle time in meristem of leaves exposed to chilling was associated with the up-regulated expression of cell cycle inhibitors such as KPRS. Similarly, the EL2 gene from rice (Orysa;E2L) was induced to transcript by low temperature. Orysa;E2L is a plant specific inhibitor of CDK that inhibit CDKA1 activation during G1/S transition through direct or indirect binding to CYCD (Peres et al., 2007). In addition, cold stress stimulates the expression of the transcription factor OsMYB3R2, which increases the expression of several G2/M-specific genes such as *OsCYCB1*. Furthermore, *OsCYCB1* overexpression increased resistance to cold stress (Ma et al., 2009). Moreover, many other cold-responsive genes have been identified in *T. salsuginea* and rice by RNA-sequencing (Zhou et al., 2014; Liu et al., 2016; Wang et al., 2017). The *T. salsuginea* gene *FILAMENTOUS TEMPERATURE-SENSITIVE H8* was shown to alleviate cold-induced photoinhibition (Liu et al., 2016). In rice (*Oryza sativa*), *LOW-TEMPERATURE RESPONSE PROTEIN KINASE 1* contributes to cold resistance by regulating cytoskeletal rearrangement. Meanwhile, phytochrome B diminishes cold tolerance by modulating the expression of *DEHYDRATION-RESPONSIVE ELEMENT-BINDING 1* and unsaturated fatty acid content (Liu et al., 2012; Yang et al., 2013; Zhou et al., 2014; He et al., 2016). Ectopic expression of the above genes increases the ability of crops to resist cold temperatures, although the underlying mechanisms are unclear. It’s of interest to investigate whether the different stress responsive genes work in the same signal pathway.

Put together, plants adjust to high-temperature and low-temperature *via* regulating the expression of different cell cycle machinery components. The fact that plants have larger number of cyclins compared with other eukaryotes may indicate cyclins function in stress response or perception to some degree. Most studies have revealed that temperature halt cell cycle progression through decrease or increase the transcript levels of related genes, however, we should take posttranslational modification of CDKs into consideration. Despite all of this, additional studies are needed to clarify the molecular basis for cell cycle regulators in response to hot or cold conditions.

#### Drought Stress

Drought stress is one of the abiotic stresses that limit plant growth and crop yield. Drought induces physiological changes in plants that enable survival, such as stomatal closure, perturbation of cell growth, and inhibition of photosynthesis. These processes involve the activation or repression of transcription factors, protein kinases, and metabolism-related enzymes. Here, we focus on the effect of drought on cell cycle proliferation and cell expansion. The growth of an organ is roughly divided into two stages: the cell proliferation stage, in which the cell number increases; and the cell expansion stage, in which cells expand to their final size. In *Arabidopsis* and sunflowers, water deficit affects both cell division and cell expansion. The reduced cell number in sunflower leaves was owing to the arrested G1/S transition. Progression to S phase is mediated by the activity of cyclin-dependent kinase A (CDKA). We can speculate that drought damages CDKA activity more or less. In wheat seedlings subjected to mild water stress, leaf elongation rate and mitotic activity were reduced in mesophyll cells due to decreased activity of CDKA1, which is required for entry into mitosis. This was accompanied by downregulation of cyclins and the accumulation of cells in G1 or G2 phase (Schuppler et al., 1998). Similarly, in maize leaves, cell division and CDKA1 activity was decreased in response to a mild water deficit, resulting in the inhibition of endosperm development. However, the amount of CDKA1 protein remained unaffected suggesting that posttranslational regulation of CDKA1 was responsible (TL and BA, 2001). In another drought-tolerant *Brachypodium* leaves, cell expansion is affected while cell proliferation is not, which is opposite to *Arabidopsis* and maize. The molecules response to drought stress strongly dependent on the developmental stage. In the leaf’s proliferation zone, drought stress had no effect on the expression of genes related to cell division. By Affymetrix tiling array analysis, the researchers identified numerous drought-responsive genes which were mainly expressed in the mature leaf zone. In summary, our current understanding of stress-regulated growth is still very fragmentary, partly because studies combining detailed growth analysis and molecular characterization of growing tissues are relatively scarce. Molecular characterization of the stress responses of growing tissues has to be investigated by further step. Broadening our knowledge of the mechanisms underlying growth reduction under stress is an important prerequisite to further improve crop productivity.

### Conclusion and Perspectives

**Figure 2:**
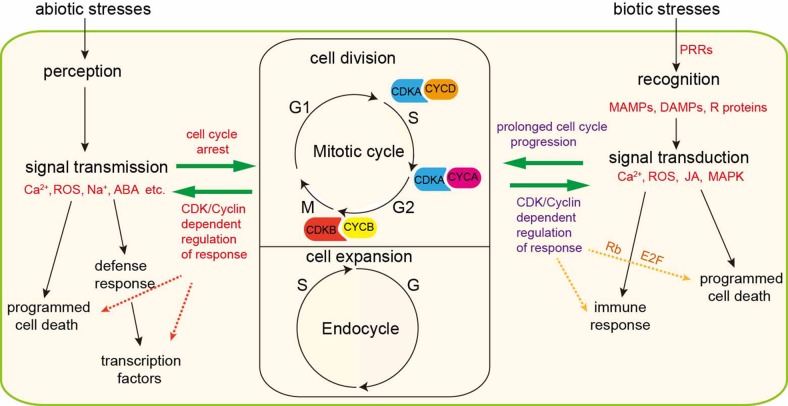
Interactions between stress/response signaling cascades and cell cycle regulation. Plant growth depends on cell division and expansion. Upon biotic stress, cell surface pattern recognition receptors (PRRs) recognize conserved microbe-associated molecular patterns (MAMPs) or damage-associated molecular patterns (DAMPs) or resistant (R) proteins and then transduce primary signal to secondary signal molecules including Ca^2+^ flux, ROS, jasmonates (JA) and MAPK and eventually initiate immunity. Immune response induces prolonged cell cycle progression or programmed cell death. Overexpression of CDKs or cyclins could enhance immune responses. Besides, G1-S phase checkpoint proteins Rb and E2F engaged in immune-related programmed cell death. Similarly, under abiotic stress conditions, plant cells sense and percept the signals and transmit them to downstream signal molecules, such as Ca^2+^, Na^+^, ABA, and ROS. These signaling cascades halt cell cycle progression through inhibiting the transcription of CDK/cyclins-related genes. Manipulating the level of CDK or cyclins could change the defense response abilities. Severe abiotic stresses trigger programmed cell death.

As sessile organisms, plants are frequently challenged as their environment changes during their lifetime. The ability of plants to perceive and adapt to these changes facilitates survival and production. Similarly, both biotic stress and abiotic stress perturb cell cycle progression and, at worst, lead to programmed cell death. Plants have evolved multiple mechanisms to defense against stresses. In brief, perception of biotic and abiotic stress signals activates signaling cascades that trigger ion fluxes, kinase cascades, reactive oxygen species production, and accumulation of hormones, such as abscisic acid (ABA) and jasmonic acid (JA). Theses signaling molecules suppress the activities of CDKs *via* controlling the expression level of cyclins or regulating the posttranslational modification of CDKs and, as a consequence, arrest the cell cycle or even exit cell cycle. This review highlights the role of cell cycle modulation in the adaption of plants to biotic and abiotic stresses and discusses the associated mechanisms proposed by recent studies (**Figure 2**). Even though the existing studies indicate that CDKs and cyclins are important for the detection and adaptation to stress by plants, there still exist many outstanding questions regarding the relationship between plant defense responses and cell cycle kinetics. For instance, it is unclear whether cyclins are directly involved in sensing stresses and whether the cyclin molecules differ in sensing abiotic stress and biotic stress. Additionally, plant proteins that detect stress and transmit signals to delay cell cycle progression remain to be identified. Moreover, it’s unknown why elevating the transcription level of cyclins or manipulating CDK activities could, to some extent, increase the ability of plants to defense against stresses. Single cell RNA sequencing approach, high-throughput, genome-wide transcriptome and proteomic technologies will help us to identify new genes and proteins involved in stress response and then broad our knowledge about cell cycle regulation and response adaption. Fully understanding the molecular signal network which regulate stress response and cell growth is the prerequisite for researchers to elevate crop plants’ resistance to stresses and improve crop plants yield.

## Author Contributions

FQ and FZ wrote the manuscript and FQ critically revised the manuscript. All authors approved the final version of the manuscript.

## Conflict of Interest

The authors declare that the research was conducted in the absence of any commercial or financial relationships that could be construed as a potential conflict interest.
